# Efficacy and Safety of 10‐Day Minocycline Twice Daily in Bismuth‐Containing Quadruple Therapy as the First‐Line Treatment of *Helicobacter pylori* Infection: A Prospective Single‐Arm Study

**DOI:** 10.1002/jgh3.70233

**Published:** 2025-07-28

**Authors:** Peiwei Li, Yan Li, Yan Chen, Cheng Fang, Qin Du, Yuehua Han

**Affiliations:** ^1^ Department of Gastroenterology Second Affiliated Hospital, Zhejiang University College of Medicine Hangzhou China

**Keywords:** amoxicillin, first‐line therapy, *Helicobacter pylori*, infection, minocycline

## Abstract

**Background:**

Tetracycline has limited clinical application in *Helicobacter pylori* treatment because of difficulty in obtaining and increased adverse reactions. As a semisynthetic tetracycline, minocycline has demonstrated good potential for eradicating *H. pylori* infection. This study aimed to evaluate the efficacy and safety of 10‐day minocycline‐based quadruple therapy for *H. pylori* first‐line treatment.

**Methods:**

In this prospective trial, treatment‐naïve adults with *H. pylori* infection received eradication therapy with rabeprazole 10 mg, minocycline 100 mg, amoxicillin 1000 mg, and bismuth potassium citrate 220 mg each given twice a day for 10 days. The primary outcome was the eradication rate. The secondary outcome was adverse effects. Eradication was confirmed by a negative urea breath test at least 6 weeks after the end of therapy.

**Results:**

A total of 133 patients were included in the study. All of the patients completed the course of medication. We found that 10‐day minocycline‐amoxicillin quadruple therapy achieved an eradication rate of 83.5% (111/133, 95% CI 80.3%–86.7%) in intention‐to‐treat analysis and 90.2% (111/123, 95% CI 87.6%–92.8%) in per‐protocol analysis. The treatment‐emergent adverse events (TEAEs) were 15% (20/133), with the most common adverse event being dizziness (14/133, 10.5%). No severe adverse event was observed.

**Conclusions:**

Ten‐day minocycline‐amoxicillin twice daily in bismuth‐containing quadruple therapy appears to be effective and safe for naïve *H. pylori* patients.

## 1 Introduction

*Helicobacter pylori* is one of the most prevalent pathogens in humans, with approximately 4.4 billion people infected globally, although a declining trend of *H. pylori* prevalence was indicated [1]. It is a leading cause of many gastric diseases, including chronic gastritis, peptic ulcer, and gastric cancer, and studies also suggested that *H. pylori* increases the risk of many extra‐gastrointestinal diseases, such as cardiovascular diseases [2, 3]. Infected family members may be the main source of *H. pylori* transmission, with a higher family‐based infection rate (71.1%), so we must pay more attention to the social and economic burden due to *H. pylori* infection [4, 5].

*H. pylori* eradication treatment is essential for decreasing the risk of its related diseases, but the eradication rate is declining with the increasing resistance to antibiotics, such as metronidazole, levofloxacin, and clarithromycin [6]. Antibiotic resistance rates of *H. pylori* kept at a higher level in Zhejiang Province [7]. In China, current guidelines have recommended first‐line *H. pylori* eradication regimens that consist of a proton‐pump inhibitor (PPI), bismuth, and two antibiotics (such as tetracycline, metronidazole) especially in regions with high antibiotic resistance [8]. However, tetracycline is difficult to obtain in China, and the high frequency of adverse events often results in limited clinical applicability of this recommendation [9]. The use of other drugs to replace tetracycline to effectively eradicate *H. pylori* infection has become one of the hot research directions in this field.

Minocycline is a semisynthetic tetracycline with a broad antimicrobial spectrum and a better bactericidal activity than tetracycline against many pathogenic bacteria [10, 11]. Moreover, the half‐life of minocycline is long, and it needs to be taken orally only once or twice daily; thus, it has a better compliance [11]. Minocycline has been widely used in the treatment of infectious diseases in clinical practice, and resistance of *H. pylori* to minocycline is rare, which is similar to tetracycline [12, 13]. Several trials evaluated a 14‐day minocycline‐containing regimen, combining minocycline plus metronidazole or amoxicillin as first‐line regimens for *H. pylori* eradication, and found that the efficacy was satisfactory with good compliance and safety [10, 12, 13, 14, 15]. Few studies have explored the first‐line 10‐day minocycline‐based quadruple therapy in *H. pylori* patients.

In the current study, we performed a prospective study to assess the efficacy of 10‐day minocycline‐amoxicillin quadruple therapy in first‐line *H. pylori* eradication.

## 2 Materials and Methods

### 2.1 Study Design

This study was a prospective, single‐center trial performed between 2021 and 2022 at the Second Affiliated Hospital of Zhejiang University, School of Medicine. The trial was approved by the Ethics Committee of the hospital, and written informed consent was obtained from all the subjects. It was registered in ClinicalTrials, and the registration number was NCT04923113. The study was also conducted in accordance with the Declaration of Helsinki.

### 2.2 Eligibility Criteria

Consecutive outpatients with positive ^13^C‐urea breath tests aged 18–65 with no history of treatment were eligible for inclusion in this trial. At baseline, all patients underwent stool antigen tests, esophagogastroduodenoscopy, and gastric biopsies (from the antrum and corpus) were obtained for histology. *H. pylori* infection was determined by a positive result of ^13^C‐UBT (4% cutoff values; CNNC Headway Biotechnology Co. LTD, Shenzhen, China) [16] and at least one of the two tests (histology and stool antigen test). Kimura‐Takemoto Classification (KTC) was used for atrophy detection [17]. The criteria for KTC were as follows: (1) C1, the atrophy confined in the antrum; (2) C2, the atrophy exceeded the incisura angularis but confined in the lesser curvature; (3) C3, the atrophy was in the lesser curvature and did not exceed the cardia; (4) O1, the atrophy extended to the cardia and the atrophic border was between the lesser curvature and the anterior wall; (5) O2, the atrophic border was in the anterior wall; (6) O3, the atrophic border was between the anterior wall and the greater curvature; C0 meant those without atrophy.

The exclusion criteria were: (1) previously treated for *H. pylori* ; (2) pregnancy or lactation; (3) previous gastric surgery; (4) presence of significant clinical diseases or malignancy; (5) use of antisecretory drugs, antibiotics or bismuth within the past 4 weeks; (6) allergy to any of the research drugs. All subjects in this study signed written informed consent forms before enrollment.

### 2.3 Intervention

Patients were treated with rabeprazole(Eisai Co. Ltd., Tokyo, Japan) 10 mg, minocycline (Hanhui Pharmaceutical Co. Ltd. Hangzhou, China) 100 mg, amoxicillin (Bright future Pharmaceutical Company, HongKong, China) 1000 mg, and bismuth potassium citrate (Zhendong Pharmaceutical Company, Jinzhong, China) 220 mg twice daily for 10 days. This was an open‐label study; physicians and subjects were aware of the treatment received. The technicians who performed ^13^C‐UBT, histology, or stool antigen test were blinded to treatment. In this study, rabeprazole and bismuth were given 30 min before morning and evening meals; minocycline was given 30 min after morning and evening meals; amoxicillin was taken with morning and evening meals. Written informed consent was obtained from all of the patients before enrollment. All subjects were informed of the drug administration times, possible adverse events, and how to report adverse events. Treatment‐emergent adverse events (TEAEs) were recorded throughout the study. TEAEs were classified as mild (not interfering with daily routine), moderate (affecting daily routine), severe (markedly affecting daily routine and discontinuation of medications), and serious (death, hospitalization, disability, or requiring intervention to prevent permanent damage). Compliance was defined as good if the subjects took above 80% of the drugs in the regimen during the consecutive 10 days.

### 2.4 *H. pylori* Isolation and Stool Antigen Test

We collected two gastric biopsy specimens (one antral and one corpus) to isolate *H. pylori* strains. *H. pylori* isolates were identified by the routine hematoxylin–eosin (HE) method. Moreover, stool samples were collected from all participants and were analyzed using a monoclonal EIA stool antigen test. The samples were tested in a laboratory where investigators were blinded to the *H. pylori* status of the patients.

### 2.5 Outcomes Assessment

The primary outcome was the eradication rate of *H. pylori* infection in the treatment group. *H. pylori* eradication was determined by ^13^C‐UBT at least 6 weeks after completion of therapy. Eradication was defined as a negative urea breath test (< 4‰; 4‰ as the cutoff value). The secondary outcomes were the frequency of overall adverse events and the compliance rate.

### 2.6 Statistical Analysis

According to the previous study, we set the reference eradication rate as 0.926 [10], and we set the expected eradication rate of the current study as 0.85 basing on data of our center. We set the statistical power at 80% (1 − β) and set the significance level at 5% using a two‐sided test. The calculated sample size was131, accounting for a 10% loss to follow‐up rate. Eradication rates were evaluated using intention‐to‐treat (ITT) and per‐protocol (PP) analysis. All subjects were included in the ITT analysis, while those who did not follow the study protocol were not included in the PP analysis. Patients without follow‐up UBT were regarded as treatment failures in the ITT analysis. Characteristics of the population were performed using descriptive statistics. Chi‐square test and variate analysis were performed to evaluate significant predictive variables for *H. pylori* eradication. *p* < 0.05 was defined as statistically significant, and all analyses were conducted using SPSS v.21 Statistics program.

## 3 Results

### 3.1 Baseline Characteristics of the Study Group

**FIGURE 1:**
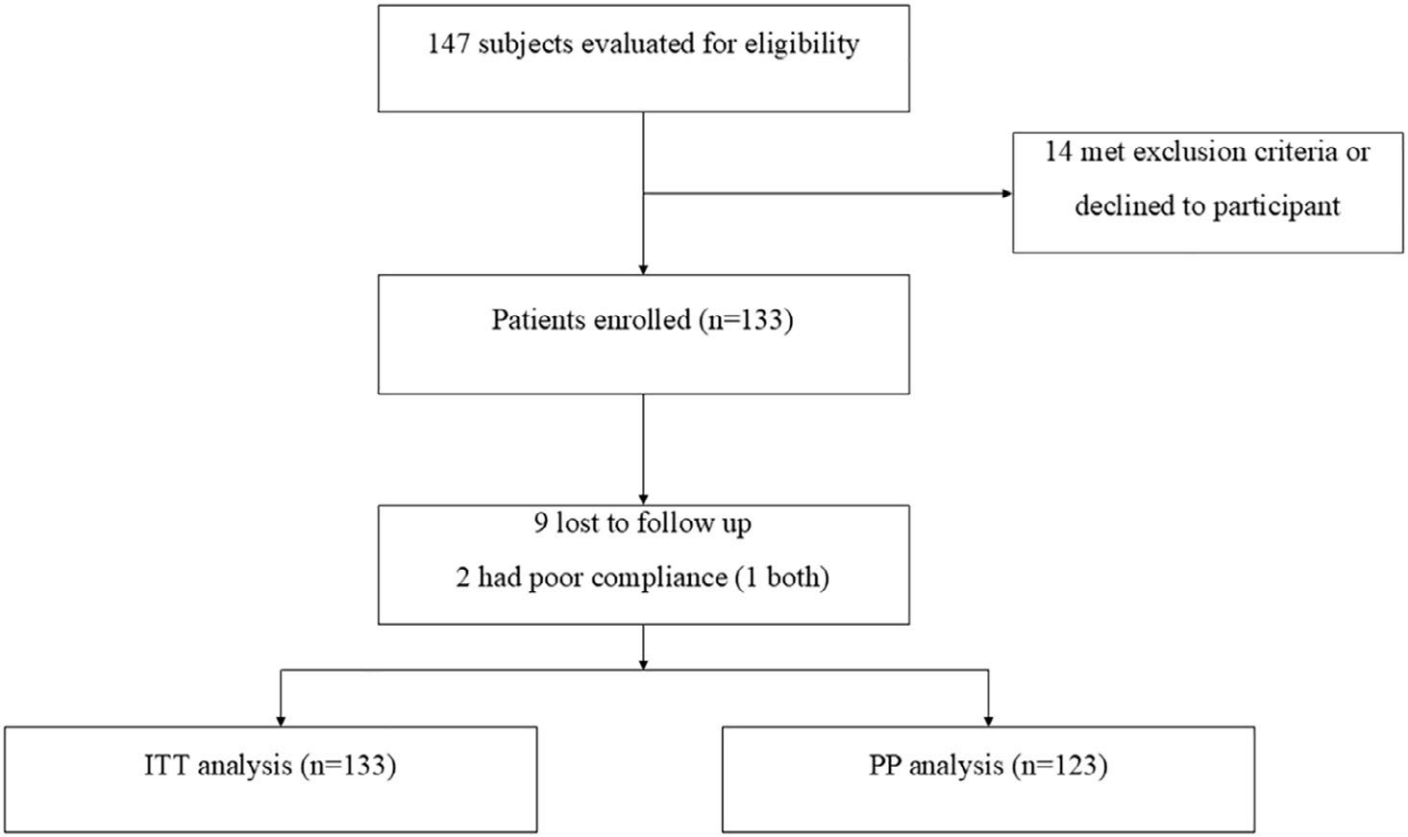
Flow diagram of the study.

A total of 147 *H pylori*‐infected patients were evaluated for eligibility, and 14 met exclusion criteria or declined to participate and were excluded. Finally, 133 patients were enrolled in the current analysis. The flow of this trial is demonstrated in Figure 1. The demographic and clinical characteristics of the enrolled population are summarized in Table 1. The mean age was 49.1, and 71 were females (53.4%) (Table 1). Overall, 9 patients were lost to follow‐up UBT, and 2 patients had poor compliance (1 both had poor compliance and lost to follow up), which were defined as treatment failure in the ITT analysis and were excluded from the PP analysis. Thus, a total of 123 patients were analyzed in the PP analysis.

**TABLE 1:** Baseline characteristics of the included patients.

	Minocycline‐containing quadruple therapy group
Age (mean ± SD)	49.1 ± 10.6
Gender (M/F)	62/71
PG I	69.75 ± 37.7
PG II	17.3 ± 13.0
Atrophy in gastroscopy	87 (65.4)
Number of patients in ITT analysis	133
Number of patients in PP analysis	123

### 3.2 *H Pylori* Eradication Rates

As shown in Table 2, the eradication rates for minocycline were 83.5% (111/133, 95% CI 80.3%–86.7%) in the ITT analysis and 90.2% (111/123, 95% CI 87.6%–92.8%) in the PP analysis, respectively.

**TABLE 2:** Eradication rate of the included patients.

Eradication rate	
ITT analysis	111/133 (83.5%, 95% CI 80.3%–86.7%)
PP analysis	111/123 (90.2%, 95% CI 87.6%–92.8%)

Abbreviations: CI, confidence interval; ITT, intention‐to‐treat; PP, per‐protocol.

The eradication rate was significantly higher in males than in females (97.0% vs. 82.1%, *p* = 0.006) (Table 3). Moreover, patients with PGI/II less than 3 had a higher eradication rate (93.3%) than those with high PGI/II level (84.9%) (*p* = 0.028) (Table 3). In the multivariate analysis, gender was significantly associated with eradication rate (OR = 0.112, 95% CI 0.013–0.982, *p* = 0.048), and no significant association between age, gastric atrophy, pepsinogen, and eradication rates was observed (Table 3).

**TABLE 3:** Risk factors for eradication failure.

Subgroups	Eradication rate	*p*
Gender		0.006
Male	97.0%	
Female	82.1%	
Age		0.486
≥ 50	92.1%	
< 50	88.3%	
Atrophy		0.282
Yes	92.6%	
No	86.1%	
PGI/II		0.028
≥ 3	84.9%	
< 3	93.3%	
Multiple analysis	OR and 95% CI	*p*
Gender	0.112 (0.013–0.982)	0.048
Age	1.578 (0.262–9.503)	0.618
Atrophy	1.512 (0.260–8.801)	0.645
PGI/II	0.469 (0.098–2.247)	0.343

Abbreviations: CI, confidence interval; OR, odds ratio.

### 3.3 Adverse Events

Adverse events were observed in 20 patients (15.0%); dizziness, nausea, fatigue, and vomiting were the most common adverse events (Table 4). No serious adverse events were observed, and no patients discontinued the treatment because of adverse events. All adverse events disappeared after cessation of treatment.

**TABLE 4:** Adverse events of the included patients.

Adverse events	
Total	20 (15.0%)
Dizziness	14 (10.5%)
Abdominal discomfort	6 (4.5%)
Fever	1 (0.75%)
Nausea and vomiting	2 (1.5%)
Diarrhea	1 (0.75%)

## 4 Discussion

Our study demonstrated that bismuth‐containing quadruple therapy with minocycline and amoxicillin has good efficacy as a first‐line treatment of *H. pylori* infections, with a satisfactory eradication rate of 83.5% for ITT analysis and 90.2% for PP analysis. Furthermore, bismuth‐containing quadruple therapy comprising minocycline and amoxicillin was well‐tolerated by patients, with a low rate of adverse events (15.0%), and no serious adverse events were observed.

The antibiotic resistance of *H. pylori* has become more serious in recent years. Our previous report found that the resistant rate was highest for metronidazole (70.0%), followed by clarithromycin (31.7%) and levofloxacin (29.5%), while the resistant rate was low for tetracycline (1.6%) and amoxicillin (0%) [7]. Thus, it is difficult to empirically choose the eradication regimens in clinical practice. The Chinese consensus recommends bismuth quadruple therapy with amoxicillin or a tetracycline as the first‐line treatment for *H. pylori* , especially in areas with high clarithromycin resistances such as Zhejiang Province [8]. However, the feasibility of this therapy is challenged in China due to the restricted accessibility to tetracycline [18]. Interestingly, studies indicate a difference in resistance rates between males and females. For instance, one study has shown that female *H. pylori* patients exhibit significantly higher resistance rates to clarithromycin and levofloxacin compared to males [19]. This may partially explain the observed disparity in eradication rates between genders for the current study. However, as the study did not perform antimicrobial susceptibility testing, it cannot fully elucidate the reasons why the eradication rate was lower in females than in males.

As a semisynthetic tetracycline, minocycline has a similar antibiotic mechanism to tetracycline. It can specifically bind to the 30S subunit of the bacterial 70S ribosome, thus preventing peptide chain extension and blocking the synthesis of protein. The bactericidal activity of minocycline has been suggested to be higher than tetracycline in many pathogenic bacteria [10], and it has a longer half‐life, can be taken twice daily, and the compliance is better [11, 15]. In addition, the primary resistance rate of *H. pylori* to this drug is rare, and secondary resistance is not prone to occur in clinical practice [10, 20].

Bismuth‐containing quadruple therapy consisting of minocycline and amoxicillin is one of the treatment options recommended by studies, which supported the use of minocycline as a potent alternative to tetracycline in *H. pylori* eradication. The current analysis further provided evidence on this issue. Most of the studies assessed 14‐day minocycline‐containing quadruple therapies, while few studies evaluated 10‐day minocycline‐containing therapies in *H. pylori* treatment [12, 13, 14, 15]. The RCT results of Zhang et al. suggested that the eradication efficacy of 14‐day minocycline–amoxicillin quadruple therapy was satisfactory (85.7% in ITT analysis and 89.5% in PP analysis), which was higher than minocycline–metronidazole therapy (77.1% in ITT analysis and 84.3% in PP analysis) [15]. Moreover, the rate of adverse events was higher for patients receiving high‐dose metronidazole, especially taste distortion, nausea, and anorexia [21]. Thus, minocycline–amoxicillin quadruple therapy might be a better choice.

Safety is also an important issue in bismuth‐containing quadruple therapy. In the current study, the overall rate of adverse events was 15.0%. Previous studies reported that about 15%–20% of patients receiving minocycline would experience dizziness, which was due to the effect of minocycline on the vestibule [11]. This side effect in most patients was mild to moderate, and few patients could not tolerate it. This result confirmed the safety of bismuth‐containing quadruple therapy comprising minocycline and amoxicillin. In the current study, the rate of dizziness was 10.5% (14/133), and no patient stopped therapy because of adverse events.

Frequent dosing results in regimen complexity, which may lead to poor patient compliance. The three times daily dose is also difficult to associate with a meal, further adding to the complexity of the regimen. Meanwhile, reducing drug dosage can also help reduce the cost of treatment. A simpler and more affordable medication regimen can reduce patient's non‐compliance to therapy, especially in areas with high clarithromycin resistances. The current study supported the 10‐day minocycline‐amoxicillin quadruple therapy in first‐line *H. pylori* eradication. The results enriched the optimal selection of clinical eradication regimens and would be helpful in eradicating *H. pylori* infection for patients living in areas with high antibiotic resistance and tetracycline inaccessible.

There are also some limitations in the current study. First, it was a single‐center study, and further trials in multiple centers are required. Second, bismith is available only in some countries. Third, the efficacy of 10‐day minocycline‐amoxicillin quadruple therapy was not compared with other therapy, such as vonoprazan‐minocycline or 14‐day minocycline‐amoxicillin regimen. Moreover, antimicrobial susceptibility testing was not performed in the current study. Therefore, further studies would be required.

In summary, 10‐day minocycline‐amoxicillin quadruple therapy can be a choice for treatment‐naïve *H. pylori* patients with relatively good eradication rates and safety. These results suggest the usefulness of the regimen as a potential first‐line therapy for patients with *H. pylori* infection.

## Conflicts of Interest

The authors declare no conflicts of interest.

## Data Availability

The data that support the findings of this study are available on request from the corresponding author. The data are not publicly available due to privacy or ethical restrictions.
